# Assessment of Periodontal Status Based on Carotid Artery Intima Media Thickness

**DOI:** 10.3290/j.ohpd.a44036

**Published:** 2020-07-04

**Authors:** Krishna N. Nitya, Dolar Doshi, Suhas Kulkarni, Madupu Padma Reddy, Adepu Srilatha, Dantala Satyanarayana

**Affiliations:** a Senior Lecturer, Department of Public Health Dentistry, Panineeya Institute of Dental Sciences and Research Centre, Telangana, India. Collected the data, clinical examination, wrote the paper.; b Assistant Professor, Department of Public Health Dentistry, Government Dental College and Hospital, Hyderabad, Telangana, India; Panineeya Institute of Dental Sciences and Research Centre, Telangana, India. Study idea and hypothesis; wrote and proofread the paper.; c Head of Department, Department of Public Health Dentistry, Panineeya Institute of Dental Sciences and Research Centre, Telangana, India. Conceived and designed the analysis.; d Professor, Department of Public Health Dentistry, Panineeya Institute of Dental Sciences and Research Centre, Telangana, India. Conceived and designed the analysis.; e Reader, Department of Public Health Dentistry, Panineeya Institute of Dental Sciences and Research Centre, Telangana, India. Statistical evaluation.; f Reader, Department of Public Health Dentistry, Panineeya Institute of Dental Sciences and Research Centre, Telangana, India. Contributed substantially to discussion.

**Keywords:** atherosclerosis, carotid artery intima media thickness, periodontal disease, India, oral hygiene

## Abstract

**Purpose:**

Atherosclerosis is a devastating disease worldwide since it is the most frequent cause of myocardial infarction, stroke, renal failure, peripheral vascular disease and perhaps dementia. There is a well-documented evidence supporting the association between clinical/subclinical atherosclerosis and periodontitis. Carotid intima media wall thickness (CIMT) is a histopathologically validated marker of atherosclerosis. This study’s purpose was to assess periodontal status based on carotid artery intima media thickness.

**Materials and Methods:**

A cross-sectional study was carried out among subjects who visited the Care Hospital, Nampally Hyderabad for CIMT test. Oral hygiene status was evaluated using Simplified Oral Hygiene Index and periodontal health status was measured using modified World Health Organization (WHO) Oral Health Assessment form, 1997. The data was analysed using Statistical Package for Social Sciences (SPSS) version 21.0. The proportions and mean scores were compared using chi-square test, Mann–Whitney U test and analysis of variance (ANOVA). Logistic regression analysis determined the relationship between periodontitis, as an independent variable and other variables with CIMT. P < 0.05 was considered statistically significant.

**Results:**

A total of 600 individuals were classified based on CIMT thickness ≤ 1 mm (292; 48.6%) and CIMT > 1 mm (308; 51.3%) according to variables. Significantly higher mean scores were observed for all oral parameters among subjects with CIMT > 1 mm aged > 45 years and among males (p ≤ 0.05*). Logistic regression analysis showed that increasing age group, ie,> 45 years (OR 3.5), males (OR 2.02), university education (OR 2.99), no history of previous dental visit (OR 3.71); and visit ≥ 1 year (OR 0.76) and previous history of tobacco (OR 1.13) and alcohol use (OR 1.65), poor OHI-S (OR 8.00), Community Periodontal Index (CPI) with Code 3, 4 (OR 4.41) and loss of attachment (LOA) with Code 2 (OR 3.05) and Code 3 (OR 5.80) had significantly higher odds among individuals with subjects with CIMT > 1 mm compared to their counterparts (p ≤ 0.05*).

**Conclusion:**

The results of the study concluded that periodontal disease and poor oral hygiene were more severe among the subjects with CIMT > 1 mm. To halt the progression of increasing CIMT, preventive oral health programmes need to be integrated in the cardiac setting with established dental referral which can bring out positive health behaviours.

Identification of patients at risk for heart attack and stroke is of great concern for a physician and for patients themselves. Atherosclerosis is a devastating disease worldwide since it is the most frequent cause of myocardial infarction, stroke, renal failure, peripheral vascular disease and perhaps dementia. In many parts of the world, it is the most common cause of congestive heart failure.^22,28–30^ Atherosclerosis usually does not cause symptoms until middle or older age, but as the arterial narrowing becomes severe, it can obstruct blood flow and cause pain. This condition usually starts in the vascular intima, progressing to the medial arterial wall. Thickening of the arterial wall due to deposition of lipid and glycol components is associated with chronic inflammation around the vessel which causes proliferation of fibres known as atherosclerotic plaque. The worst happens when plaques suddenly rupture, allowing blood to clot inside an artery, in the brain, thus causing a stroke in the heart, eg, a heart attack.^1,16,17,19,27,30,37^

Currently, several imaging tests can detect the presence of atherosclerotic plaque in the carotid and coronary arteries. While some tests (eg, angiography, intravascular ultrasound) involve a small dose of radiation, the Carotid Intima Media Thickness test (CIMT) uses sound waves (ultrasound – no radiation) and is uniquely suited to detect not just calcific (hard) plaques but also subtle soft plaques in the carotid arteries.^7,8,10,12,13,33,36^ CIMT is a non-invasive technique which measures the thickness of the inner two layers of the carotid artery (intima and media) at near and far walls of the common carotid artery (CCA), bifurcation, and internal carotid artery (ICA), with the CCA being the most common site to be measured (Fig 1). The intima media thickness (IMT) of the carotid artery is a histopathologically validated marker of atherosclerosis.^5,13,33,34^

**Fig 1:**
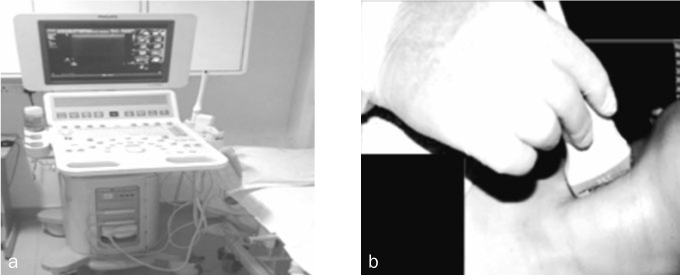
Measurement of carotid artery intima media thickness (CIMT).

Typically, normal CIMT at age 10 is approximately 0.4–0.5 mm, while this progresses to 0.7 mm to 0.8 mm or more after the fifth decade of life.^34^ CIMT above 1.0 mm is regarded as abnormal and if the IMT is above 1.2 mm, the patient is considered to be at high risk for cardiovascular diseases (Fig 2). The risk factors include high lipoprotein levels, high blood pressure, smoking, diabetes mellitus, obesity and a sedentary lifestyle and further ageing also acts as a contributing factor to increased CIMT.^20,42^

**Fig 2:**
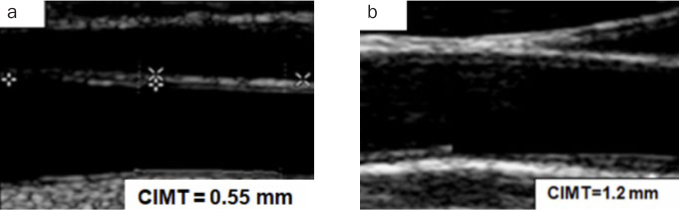
Carotid artery intima media thickness (CIMT) test values.

Since the late 1980s, several papers have linked poor oral health with cardiovascular diseases.^2–4,35^ Several studies^2,4,15,21,24,25,35,38,40,44^ have shown that oral pathogens that have been implicated in periodontal disease, like *Porphyromonas gingivalis*, *Aggregatibacter actinomycetemcomitans*, *Tannerella forsythia*, *Prevotella intermedia*, *Chlamydia pneumonia* and *Streptococcus sanguis*, also play a major role in the aetiology of atherosclerosis, causing progressive narrowing of the lumen.

A probable correlation between the severity of periodontal disease and CIMT also has been reported by Beck et al.^2^ The authors found that patients with severe periodontal disease had 1.3 times the odds of having thick carotid arterial walls ≥ 1 mm compared to those without periodontal disease. However, the relationship between periodontal disease and atherosclerosis has witnessed interesting yet contentious results ranging from strong links^15,21,24,40,44^ to absence of any causal relationship.^6,11^

With this background, the aim of the present study was to assess the periodontal status based on carotid artery IMT. Objective is to explore the association between periodontal status and carotid artery IMT based on age and gender.

## Materials and Methods

A cross-sectional study was conducted for a period of 3 months from January to March 2018 among the patients attending Care Hospital, Nampally, Hyderabad. Ethical clearance was obtained from the Institutional Review Board of Panineeya Mahavidyalaya Institute of Dental Sciences and Research Centre (PMVIDS& RC/IEC/PHD/DN/0121-16). The study was conducted in accordance with the Declaration of Helsinki.^43^ Permission to carry out the study was obtained from the authorities of Care Hospital, Nampally Branch, Hyderabad.

A pilot study was conducted among 30 individuals to check feasibility of the study and to estimate the sample size. Subjects participating in the pilot study were excluded from the final sample.

Based on the periodontal status scores, the sample size was determined as follows: with an expected proportion of 0.561, and at a confidence level of 95% and with precision of 5%, the minimum estimated sample size is 378. This was calculated by using the formula.

Inclusion criteria of the study included subjects aged 30 years and above, subjects undergoing ultrasonography to measure CIMT, patients who give written informed consent and subjects with no history of carotid artery surgery. Exclusion criteria were those subjects with history of use of antibiotics for more than 1 week in the past 6 months and subjects requiring antibiotic prophylaxis before clinical examination. Subjects who were visiting Radiology Department of Care Hospital, Nampally for Carotid Intima Media Thickness (CIMT) test on the day of the examination were included in the study. A schedule for the survey was prepared prior to data collection. The survey was conducted within the working hours of the hospital, as per the time allotted by the authorities of the respective hospital.

The clinical examination of all the subjects was done by a single pretrained, precalibrated examiner to limit intraexaminer variability. The training and calibration was done in the Department of Public Health Dentistry under the guidance of a senior faculty. The recorder was also pretrained in the department. The examiner was assisted by a pretrained clerk. Before the survey, the recording clerk was told about the terms and coding systems and was trained in the Department of Public Health Dentistry. At the end of each day, all the filled proformas were reviewed by the investigator for accuracy and completeness of recordings.

The examination was done in the aforementioned hospital by using mouth mirror, no.5 explorer (Shepherd’s Crook) and CPI probe under adequate natural light (Type III examination). The study subjects were made to sit on a chair, with the examiner standing behind or in front. The dental instruments were placed on a table within easy reach of the examiner.

Survey tools included: demographic information (age, gender, educational qualification, marital status); CIMT test value; history of systemic conditions (diabetes, hypertension, etc); oral hygiene practices (previous visit to dentist, method of cleaning and frequency of cleaning); and deleterious oral habits (tobacco and alcohol habits).

In clinical oral examination, the oral hygiene status was assessed using Simplified Oral Hygiene Index (OHI-S) by John C. Greene and Jack R. Vermillion (1964).^14^ The periodontal status was assessed using the CPI and loss of attachment (LOA) indices based on the codes and criteria according to WHO proforma (1997).^32^

### Statistical Analysis

Statistical analyses were done using the Statistical Package for Social Sciences (SPSS version 21.0). Descriptive statistics were carried out for the demographic variables. Chi-square analysis was used to find the statistical significance of two or more variables. Analysis of variance (ANOVA), and Mann–Whitney U test were used for comparison among variables. To check the relationship between periodontitis, as an independent variable and other variables with CIMT logistic regression analysis was performed. P < 0.05 was considered statistically significant.

## Results

In the present study, a total of 600 subjects, comprising of 432 (71.8%) males and 168 (28.2%) females were examined. All the patients were classified based on CIMT thickness ≤ 1 mm (292; 48.6%) and CIMT > 1 mm (308; 51.3%) according to demographic variables. The majority of the subjects aged above 45 years (89.6%) with a habit of tobacco use had CIMT > 1 mm. Around 45.2% of the individuals had their previous dental visit wherein, 19.2% visited the dentist at intervals of between 6 months to 1 year (Table 1).

**Table 1:** Demographic distribution of the study population

Variables CIMT ≤ 1 mm		n (%)		
		CIMT > 1 mm	TOTAL	
Age groups	≤ 45	103 (35.4)	32 (10.4)	135 (22.5)
	> 45	189 (64.6)	276 (89.6)	465 (77.5)
Gender	Males	183 (62.9)	249 (80.5)	432 (71.8)
	Females	109 (37.1)	59 (19.5)	168 (28.2)
Marital status	Married	292 (100)	308 (100)	600 (100)
	Unmarried	0 (0)	0 (0)	0 (0)
Education	Primary school	42 (14.4)	59 (19.2)	101 (16.8)
	High school	38 (12.7)	90 (29.2)	128 (21.4)
	University	212 (72.9)	159 (51.6)	371 (61.8)
Previous dental visit	No	131 (45)	198 (64.3)	329 (54.8)
	Yes	161 (55)	110 (35.7)	271 (45.2)
Last dental visit	No	131 (45)	198 (64.3)	329 (554.8)
	6 months–1 year	69 (23.7)	46 (14.9)	115 (19.2)
	≥ 1 year	92 (31.3)	64 (20.8)	156 (26)
History of tobacco use	No	126 (43)	93 (30.2)	219 (36.5)
	Yes	166 (57)	215 (69.8)	381 (63.5)
History of alcohol use	No	57 (19.2)	87 (28.2)	144 (24)
	Yes	236 (80.8)	221 (71.8)	457 (76.2)
History of systemic conditions	Healthy	192 (65.6)	225 (73.1)	417 (69.5)
	Diabetes	100 (34.4)	83 (26.9)	183 (30.5)
Method of cleaning	Toothbrush and toothpaste	292 (100)	308 (100)	600 (100)
	Any other	0 (0)	0 (0)	0 (0)
Frequency of toothbrushing	Once	292 (100)	308 (0)	600 (100)
	Twice or more	0 (0)	0 (0)	0 (0)
Total		292	308	600 (100)

Majority of the study subjects had fair scores for Debris Index-Simplified (DI-S) (455; 75.8%), Calculus Index-Simplified (CI-S) (325; 54.2%) and total Simplified-Oral Hygiene Index (OHI-S) (344; 57.3%). A statistically significant difference in all the oral hygiene parameters was observed with population having CIMT ≤ 1 mm recording good scores (p = 0.0001*) (Table 2).

**Table 2:** Distribution of the study population based on OHI-S scores

Variables CIMT ≤ 1 mm		n (%)			
		CIMT > 1 mm	P value	Total	
Debris index-simplified (DI-S)	Good	76 (25.8)	28 (9.1)	0.0001*	104 (17.3)
	Fair	216 (74.2)	239 (77.6)		455 (75.8)
	Poor	0 (0)	41 (13.3)		41 (6.8)
Calculus index- simplified (CI-S)	Good	27 (8.9)	0 (0)	0.0001*	27 (4.5)
	Fair	196( 67.4)	129 (41.9)		325 (54.2)
	Poor	69 (23.7)	179 (58.1)		248 (41.5)
Simplified oral hygiene index (OHI-S)	Good	75 (25.8)	0 (0)	0.0001*	75 (12.5)
	Fair	196 (67.4)	148 (48.1)		344 (57.3)
	Poor	21 (6.9)	160 (51.9)		181 (30.2)

*p ≤ 0.05 statistically significant.

A statistically significant difference (p = 0.0001*) was noted in the CPI and LOA status of the study population based on CIMT, wherein significantly higher percentage of population with CIMT > 1 mm had code 2 (160; 51.9%) and code 3 (134; 43.5%) for CPI. Meanwhile, around 23.2% of population with CIMT ≤ 1 mm had code 4, ie, pocket 6 mm or more. On the contrary, overall the LOA status was better among subjects with CIMT > 1 mm with 42.2% recording code 0 (130; 42.2 %). Likewise, significantly higher population of individuals with CIMT ≤ 1 mm had LOA codes 1, 2 and 3 compared to those with CIMT > 1 mm (p = 0.0001*). (Table 3)

**Table 3:** Distribution of the study population based on CPI and LOA coding criteria

Variables CIMT ≤ 1 mm		n (%)			
		CIMT > 1 mm	P value	Total	
Community Periodontal Index (CPI)	Code 0	0 (0)	0 (0)	0.0001*	0 (0)
	Code 1	12 (4.1)	14 (4.5)		26 (43.3)
	Code 2	119 (40.7)	160 (51.9)		279 (46.5)
	Code 3	93 (31.8)	134 (43.5)		227 (37.8)
	Code 4	68 (23.2)	0 (0)		68 (11.3)
	Code X	0 (0)	0 (0)		0 (0)
	Code 9	0 (0)	0 (0)		0 (0)
Loss of attachment (LOA)	Code 0	108 (37.2)	130 (42.2)	0.0001*	238 (39.7)
	Code 1	14 (4.8)	12 (3.9)		26 (4.3)
	Code 2	86 (29.4)	80 (25.9)		166 (27.7)
	Code 3	84 (28.8)	86 (27.9)		170 (28.3)
	Code 4	0 (0)	0 (0)		0 (0)
	Code X	0 (0)	0 (0)		0 (0)
	Code 9	0 (0)	0 (0)		0 (0)

*p ≤ 0.05 statistically significant.

Based on age group, all the oral health parameters showed a statistically significant difference based on CIMT. When oral hygiene was accounted, subjects aged > 45 years and with CIMT ≤1 mm had significantly higher mean scores for DI-S (1.84 ± 0.06), CI-S (1.74 ± 0.02) and OHI-S (1.94 ± 0.49). Likewise, similar situation was observed among subjects with CIMT > 1 mm wherein, individuals aged > 45 years had significantly higher mean scores for DI-S (2.15 ± 0.36), CI-S (2.60 ± 0.01) and OHI-S (2.54 ± 0.49), respectively (p = 0.0001*). Higher statistically significant mean periodontal status scores were observed in individuals > 45 years in both groups, ie, CIMT ≤ 1 mm and CIMT > 1 mm (p = 0.0001*) (Table 4).

**Table 4:** Comparison of mean OHI-S, CPI and LOA scores based on age

Variables	Mean ± SD				P value	Mean ± SD
	CIMT ≤ 1 mm		CIMT > 1 mm			Total
	≤ 45 years	> 45 years	≤ 45 years	> 45 years		
Debris Index-Simplified (DI-S)	1.56 ± 0.49	1.84 ± 0.36	2.06 ± 0.24	2.15 ± 0.36	0.0001*	1.55 ± 0.17
Total	1.76 ± 0.09		1.10 ± 0.38			
Calculus Index-Simplified (CI-S)	1.73 ± 0.01	1.96 ± 0.18	2.37 ± 0.49	1.60 ± 0.48	0.0001*	1.01 ± 0.10
Total	1.89 ± 0.01		1.35± 0.01			
Simplified Oral Hygiene Index (OHI-S)	1.56 ± 0.49	1.94 ± 0.49	2.28 ± 0.45	2.54 ± 0.49	0.0001*	2.18 ± 0.02
Total	1.58 ± 0.01		2.33 ± 0.08			
Community Periodontal Index (CPI)	1.14 ± 0.89	1.84 ± 0.79	3.10 ± 0.45	3.18 ± 0.49	0.0001*	3.13 ± 1.10
Total	1.88 ± 1.01		3.28 ± 0.12			
Loss of attachment (LOA)	0.49 ± 0.82	1.13 ± 0.24	3.04 ± 1.03	3.88 ± 0.33	0.0001*	3.01 ± 0.65
Total	1.40 ± 0.11		3.24 ± 0.09			

*p ≤ 0.05 statistically significant.

For subjects with CIMT ≤ 1 mm and CIMT > 1 mm, significantly higher mean scores were observed for all the oral parameters among males when compared to females (p = 0.0001*) (Table 5).

**Table 5:** Comparison of mean OHI-S, CPI and LOA scores based on gender

Variables	Mean ± SD				p value	Mean ± SD
	CIMT ≤ 1 mm		CIMT > 1 mm			Total
	Males	Females	Males	Females		
Debris Index-Simplified (DI-S)	1.86 ± 0.34	1.53 ± 0.50	2.33 ± 0.29	2.09 ± 0.46	0.0001*	2.00 ± 0.08
Total	1.78 ± 0.02		2.01 ± 0.13			
Calculus Index-Simplified (CI-S)	2.01 ± 0.44	1.96 ± 0.18	2.60 ± 0.02	2.47 ± 0.08	0.0001*	2.16 ± 0.01
Total	0.75 ± 0.13		2.19 ± 0.01			
Simplified-Oral Hygiene Index (OHI-S)	1.94 ± 0.34	1.86 ± 0.75	3.70 ± 0.50	2.68 ± 0.49	0.0001*	2.64 ± 0.03
Total	1.49 ± 0.02		2.21 ± 0.03			
Community Periodontal Index (CPI)	1.75 ± 0.83	1.25 ± 0.75	3.34 ± 0.45	2.98 ± 0.39	0.0001*	3.03 ± 1.12
Total	1.53 ± 0.56		3.08 ± 0.18			
Loss of attachment (LOA)	1.53 ± 0.86	1.35 ± 0.24	3.74 ± 1.03	3.18 ± 0.37	0.0001*	3.23 ± 0.13
Total	1.40 ± 0.09		3.43 ± 0.09			

*p ≤ 0.05 statistically significant.

Age and gender-wise comparison had showed that subjects aged > 45 years and males had significantly higher mean scores for both CIMT ≤ 1 mm and CIMT > 1 mm, respectively, when compared to those aged ≤ 45 years and females (p = 0.0001*) (Table 6).

**Table 6:** Comparison of Carotid Intima Media Thickness (CIMT) test mean scores based on age and gender

Variables CIMT ≤ 1 mm		Mean ± SD		P value	Mean ± SD
		CIMT > 1 mm		Total	
Age groups	≤ 45 years	0.81 ± 0.13	1.01 ± 0.12	0.0001*	1.01 ± 0.21
	> 45 years	0.94 ± 0.01	1.09 ± 0.12		
Gender	Males	0.86 ± 0.20	1.15 ± 0.21	0.0001*	1.10 ± 0.03
	Females	0.74 ± 0.30	1.01 ± 0.31		

*p ≤ 0.05 statistically significant.

When the predictors of CIMT thickness were assessed; no history of previous dental visit and positive history of alcohol use were statistically significant predictors for CIMT ≤ 1 mm. On the other hand, increasing age group, ie,> 45 years (OR 3.5), males (OR 2.02), university education (OR 2.99), no history of previous dental visit (OR 3.71); and visit ≥ 1 year (OR 0.76) and previous history of tobacco (OR 1.13) and alcohol use (OR 1.65) had significantly higher odds as compared to their respective counterparts for among those with CIMT > 1 mm (p = 0.0001*). (Table 7)

**Table 7:** Logistic regression analysis of CIMT based on variables

Variables		CIMT ≤ 1 mm		CIMT > 1 mm	
		Odds ratio	P value	Odds ratio	P value
Age groups	≤ 45 years	Ref		Ref	
	≥ 45 years	0.64 (0.51–0.81)	0.72	3.5 (1.66–4.01)	0.0001*
Gender	Females	Ref		Ref	
	Males	0.56 (0.41–1.06)	0.68	2.02 (1.06–2.31)	0.0001*
Education	Primary school	Ref		Ref	
	High school	0.41 (0.11–0.79)	0.09	0.56 (0.78–1.99)	0.09
	University	0.43(0.21–0.77)	0.07	2.99 (1.76–3.29)	0.0001*
Previous dental visit	Yes	Ref		Ref	
	No	1.70 (0.51–1.97)	0.05*	3.71 (2.51–4.07)	0.0001*
Last dental visit	6 months to 1 year	Ref		Ref	
	> 1 year	0.44 (0.13–0.59)	0.09	0.76 (0.46–1.15)	0.03*
History of tobacco use	No	Ref		Ref	
	Yes	0.74 (0.57–0.97)	0.76	1.13 (0.43–1.68)	0.03*
History of alcohol use	No	Ref		Ref	
	Yes	1.55 (1.11–2.17)	0.01*	1.65 (1.01–1.99)	0.01*
History of systemic conditions	Healthy	Ref		Ref	
	Diabetes	0.83 (0.62–1.11)	0.07	1.06 (0.37–1.68)	0.21
Method of cleaning	Toothbrush and toothpaste	Ref		Ref	
	Any other	0.0 (0.0–0.0)	0.2	0.0 (0.0–0.0)	0.2
Frequency of toothbrushing	Twice or more	Ref		Ref	
	Once	0.34 (0.14–0.67)	0.7	0.54 (0.18–0.77)	0.21

*p ≤ 0.05 statistically significant.

Based on oral parameters, it was observed that subjects with CIMT ≤ 1 mm had 1.37 times higher odds of having CPI code 3 and 4 and 1.05 times greater odds of LOA code 2. On the other hand, subjects with CIMT > 1 mm had significantly higher odds of having poor OHI-S (OR 8.00; CI 5.03–12.73), CPI Codes 3, 4 (4.41; 3.15–6.16) and LOA Code 2 (3.05;2.14–4.34) and Code 3 (5.80; 3.79–8.87) (p ≤ 0.05*) (Table 8).

**Table 8:** Logistic regression analysis of CIMT based on oral parameters

Variables Odds ratio		CIMT ≤ 1 mm		CIMT > 1 mm	
		P value	Odds ratio	P value	
OHI-S	Good	Ref		Ref	
	Fair	0.32 (0.11–0.61)	0.07	0.76 (0.61–0.94)	0.01*
	Poor	0.78 (0.03–1.13)	0.82	8.00 (5.03–12.73)	0.000*
CPI	Code 1, 2	Ref		Ref	
	Code 3, 4	1.37 (0.76–1.67)	0.05*	4.41 (3.15–6.16)	0.0001*
LOA	Code 0, 1	Ref		Ref	
	Code 2	1.05 (0.14–1.34)	0.02*	3.05 (2.14–4.34)	0.0001*
	Code 3	1.01 (0.59–1.37)	0.08	5.80 (3.79–8.87)	0.0001*

*p ≤ 0.05 statistically significant.

## Discussion

Poor oral hygiene not only increases the risk of severe periodontitis and chronic inflammation but also increases the number and virulence of periodontal pathogens that enter the bloodstream. Tonetti et al^41^ suggested a plausible biological mechanism, ie, after entry of periodontal bacteria into the circulation, multiple host inflammatory and immune responses are activated that promote the formation, maturation and exacerbation of atheroma. This proves that severe periodontitis is associated with greater thickness in the intima and media layer of the carotid arteries, leading to the narrowing and hardening of the arteries (called atherosclerosis) which subsequently results in coronary heart diseases.^7,12,17,27,36,37^ Hence, the present study focused on the relationship of periodontal disease with a non-invasive measure of atherosclerosis, ie, to assess the periodontal status based on carotid artery IMT.

Apart from the CIMT test, other tests such as ankle-brachial index (ABI), abdominal aortic diameter (AAD) coronary artery calcium score (CACS), single-photon emission computed tomography (SPECT), positron emission tomography (PET), myocardial contrast echocardiography (MCE), cardiac magnetic resonance imaging (CMRI), and cardiac computed tomography (CT) can also be used to diagnose the extent of carotid atherosclerotic vascular disease. However, CIMT has an added advantage of allowing observation of the arterial wall, the actual site of the atherosclerotic disease, rather than the lumen and can detect atherosclerotic diseases in early and asymptomatic stages.^23^

Oral hygiene was assessed using OHI-S index^14^ by John C. Greene and Jack R. Vermillion, since the criteria are clear, and examinations can be carried out quickly. The periodontal status was assessed using CPI and LOA index as per WHO codes and criteria (1997)^32^ as it is simple, reproducible and shows international uniformity.

In the present study, males had higher predominance (80.5%) of having CIMT > 1 mm. Likewise, though an existing study by Pinho et al^35^ among Portuguese adult patients attending Sao Joao Hospital showed that males had higher predominance for CIMT > 1 mm (60%), it was still less when compared to the present study. A possible reason for this could be that men are more addicted to alcohol and tobacco, which lowers the high-density lipoprotein cholesterol (HDL) in long-term use and additionally the constituents of cigarette like nicotine and carbon monoxide damage the endothelium, setting the stage for the build-up of plaque.^39^ Surprisingly, a study by Yu et al^44^ among Chinese adults attending Gucheng Hospital had only 34.6% males with CIMT > 1.2 mm.

A case–control study by Mahendra et al^26^ among 102 adult Indian patients in Chennai observed that subjects aged > 40 years had higher mean scores for OHI-S (4.95 ± 1.11) among the experimental group (cardiac group). Likewise, among Portuguese patients a higher mean percentage for dental plaque (70.8 ± 32.2) was noticed among subjects aged > 50 years and with CIMT > 1 mm.^35^ Similar to this, in the present study we observed that subjects aged > 45 years had higher mean scores for total OHI-S (2.33 ± 0.08). This could be related to their oral health behaviours in the present study, where the brushing frequency in this group of individuals was only once daily, and in addition only 35.7% had visited a dentist before. This finding emphasises the need that medical and dental communities should take necessary steps towards accurately documenting the connection between medical conditions, diseases and oral health, as supported by Chitta et al.^9^

Another remarkable observation of the study was that males with CIMT > 1 mm had higher mean scores for CPI (3.34 ± 0.45) and LOA (3.74 ± 1.03) compared to females. Similarly, a study conducted by Hayashida et al^15^ among adult patients attending Specific Health Check-up and Guidance in Japan who showed that males with CIMT > 1 mm had higher mean scores for probing depth (1.64 ± 0.60) and clinical attachment loss (2.87 ± 1.07). A practical reason cited by the American Academy of Periodontology^18^ is that men see their dentist less frequently than women, and often only when there is a serious problem. Also, they suggested that regular alcohol consumption among men can increase chances of gum disease or worsen pre-existing conditions of gum disease as the saliva, which neutralises acid in plaque, slows down when alcohol is present which thereby increases plaque and decay on the teeth and gums.

Increasing age, males, subjects with university education, subjects with no history of previous dental visit, individuals with a habit of tobacco and alcohol use and poor oral parameters such as OHI-S, CPI and LOA emerged as statistically significant predictors of increase in CIMT value > 1 mm. Similarly, a study by Beck et al^2^ among the population belonging to four US communities showed that male sex, 5-year increments of age, diabetes, hypertension, basic education and current heavy smoking were the predictors of CIMT > 1 mm. These findings bring to light the substantial role played by demographic and behavioural factors in oral and cardiac diseases.^31^

Even though the present study showed an association between periodontitis and CIMT, the study also acknowledges certain limitations. The cross-sectional study design prevents us from establishing a temporal relationship between periodontal diseases and CIMT. Investigations were done only for the clinical measures of periodontal diseasex; microbiological aspects and the important infectious markers of periodontal disease, which are more specific than clinical signs of periodontitis, have not been covered in this study due to logistic concerns. A single hospital-based study design may further limit the generalisation of the results which could be overcome by replicating the study in a homogenous group, representative of the larger national population.

## Conclusion

The results of the study concluded that periodontal disease and poor oral hygiene was more severe among the subjects with CIMT > 1 mm and in males. Other contributing factors such as tobacco and alcohol use, and oral hygiene behaviour further accentuate the risk of periodontitis. The findings from this study suggest that many patients have poor oral health, yet they lack awareness of the importance of oral health and its potential impact on progression of atherosclerosis and its outcome. In a developing and populous nation like India, limited oral health advice is provided in the cardiac/medical setting, and difficulty in accessing timely and affordable dental services further exacerbate this issue. Hence, preventive oral health programmes need to be integrated in the cardiac setting with established dental referral, which can bring out positive health behaviours.
